# slideimp: efficient imputation of DNA methylation data

**DOI:** 10.1093/bioinformatics/btag318

**Published:** 2026-05-21

**Authors:** Hung Pham, Adam P Lombroso, Esma Cansu Cevik, Hugh S Taylor, Kieran J O’Donnell

**Affiliations:** Yale Child Study Center, Yale School of Medicine, New Haven, CT 06520, United States; Department of Obstetrics, Gynecology and Reproductive Sciences, Yale School of Medicine, New Haven, CT 06520, United States; Yale Child Study Center, Yale School of Medicine, New Haven, CT 06520, United States; Department of Obstetrics, Gynecology and Reproductive Sciences, Yale School of Medicine, New Haven, CT 06520, United States; Department of Obstetrics, Gynecology and Reproductive Sciences, Yale School of Medicine, New Haven, CT 06520, United States; Department of Obstetrics, Gynecology and Reproductive Sciences, Yale School of Medicine, New Haven, CT 06520, United States; Yale Child Study Center, Yale School of Medicine, New Haven, CT 06520, United States; Department of Obstetrics, Gynecology and Reproductive Sciences, Yale School of Medicine, New Haven, CT 06520, United States; Department of Psychology, Yale University, New Haven, CT 06520, United States

## Abstract

**Summary:**

We developed slideimp, an R package that extends and optimizes K-nearest neighbor (K-NN) and Principal Component Analysis (PCA) imputation with grouped and sliding-window modes for accurate and efficient imputation of microarray and whole-genome DNA methylation (DNAm) data, respectively. Under a realistic scenario, slideimp achieved ≈12–28× faster runtime and ≈3–6× peak memory usage reduction for DNAm microarray imputation (GSE286313, EPICv2, *N* = 72) and achieved high imputation accuracy in a whole-genome DNAm dataset (*N* = 41).

**Availability and implementation:**

The code used in this study is available at https://github.com/hhp94/slideimp_paper. The R package slideimp is available on CRAN (DOI: 10.32614/CRAN.package.slideimp). Version 1.0.0 of slideimp, which was used in this study, is archived on Zenodo (DOI: 10.5281/zenodo.20029382).

## 1 Introduction

DNA methylation (DNAm), the addition of a methyl group to nuclear DNA, most commonly cytosine-phosphate-guanine (CpG) dinucleotides, is one of the most studied epigenetic modifications in humans (Aristizabal *et al.* 2020). In large-scale epidemiological analyses, DNAm is typically profiled using DNA microarrays that provide sparse (<4%) genome-wide coverage. Whole-genome bisulfite sequencing (WGBS) or Enzymatic Methyl-seq (EM-seq) describe whole-genome DNAm variation (≈30 million sites) but at a higher per-sample cost (Lister *et al.* 2009, Vaisvila *et al.* 2021).

Imputation of missing values is essential for DNAm analyses, especially for sequencing-based approaches where the total missingness rate (at 30x coverage) can range from 10% to 40% (Zou *et al.* 2018). Hidden Markov model (METHimpute) or gradient boosting methods (BoostMe) (Taudt *et al.* 2018, Zou *et al.* 2018) are used for the imputation of whole-genome DNAm data. K-nearest neighbors (K-NN) (impute), principal component analysis (PCA) (missMDA), and regression-based (methyLImp2) imputation are commonly used to impute microarray DNAm data (Troyanskaya *et al.* 2001, Josse and Husson 2016, Plaksienko *et al.* 2024).

The decreasing per-sample cost for both whole-genome and DNAm microarray analyses is facilitating the generation of increasingly large DNAm datasets, necessitating more computationally efficient yet accurate imputation methods. For microarray DNAm imputation, which involves extremely high-dimensional data where *N* ≪ *p*, *impute.knn* (impute) is limited by single-threaded execution and suboptimal default parameters, while *imputePCA* (missMDA) can be computationally inefficient. Even with the substantial increase in efficiency from parallel imputation by chromosome as implemented in methyLImp2 (Plaksienko *et al.* 2024), this approach can be computationally demanding for larger datasets. For whole-genome DNAm imputation, BoostMe is no longer actively maintained and METHimpute does not make use of between-sample information to improve accuracy. Here, we introduce slideimp, an R package that implements optimized K-NN and PCA imputation using Rcpp and RcppArmadillo (Eddelbuettel and Francois 2011, Eddelbuettel and Sanderson 2014) with a CpG-grouping mode for microarray data and a sliding-window mode for whole-genome data. slideimp’s K-NN imputation (*knn_imp*) improves accuracy with distance-weighted averaging, and enhances efficiency with OpenMP thread-level parallelization and optional mlpack’s ball tree nearest neighbor searches (Omohundro 1989, Curtin *et al.* 2023). slideimp’s PCA imputation (*pca_imp*) builds upon *imputePCA* by performing a truncated eigendecomposition on the smaller Gram matrix then recovers the sum of tail eigenvalues via the trace identity for numerical stability.

Using real-world data and simulations, we benchmark slideimp versus existing approaches for imputation of DNAm microarray data. We also show that sliding-window PCA and K-NN imputation improve whole-genome DNAm imputation accuracy with implications for derived DNAm biomarkers of aging.

## 2 Materials and methods

### 2.1 Data

For microarray DNAm data, we used the Gene Expression Omnibus GSE286313 DNAm data (*N* = 72): blood samples profiled using the EPICv2 DNAm microarrays across four cohorts of mixed sex and diverse geographical origin, spanning early to late adulthood (Zhuang *et al.* 2025). For a second dataset, we used the GSE264438 data (*N* = 581): DNAm from human cell lines and tissues profiled using the Methylation Screening Array (MSA) (Goldberg *et al.* 2025).

For whole-genome DNAm data, we used EM-seq (30× coverage) to describe DNAm in whole blood collected from 41 non-pregnant women (mean age of 32.9 years, SD of 7.1) without malignancy recruited from Yale New Haven Hospital between 2023 and 2025.

### 2.2 DNAm microarray imputation benchmarks

First, we benchmarked slideimp’s imputation efficiency using the GSE286313 data (899 653 CpGs). We compared the total runtime and peak memory usage of slideimp’s *knn_imp* and *pca_imp* against *impute.knn*, *imputePCA*, and *methyLImp2*. To benchmark parallel scaling, *knn_imp* was parallelized using OpenMP, while the other methods were run in parallel by chromosome using an increasing number of computing cores (1, 2, 4, and 8 cores), with each configuration repeated for 5 replicates.

We then compared the accuracy of K-NN and PCA imputation with and without CpG-grouping by chromosome, using Monte Carlo cross-validation (MC-CV) on both the GSE286313 and GSE264438 dataset (271 613 CpGs). For each dataset, we performed 30 independent draws, each consisting of a random subset of 400 CpGs. For every draw and each selected CpG, we masked the DNAm beta-values of 25 random participants, then re-imputed the masked values. We calculated the mean absolute error (MAE) and root mean square error (RMSE) of the masked values to compare grouped imputation (i.e. independently imputing each chromosome) versus ungrouped imputation using all CpGs.

Next, we compared K-NN and PCA imputation accuracy against *methyLImp2* using the GSE286313 chromosome 22 data (19 429 CpGs) under increasing total missing data rates from 0.5% (unmodified) to 5% by removing values at random. We then calculated the MAE and RMSE of 10 000 randomly masked and re-imputed values using MC-CV with 30 replicates.

DNAm microarray data are often used to derive estimates of epigenetic age (i.e. predicted age based on DNAm variation at “clock” CpGs). This workflow requires a beta-value matrix in which clock CpGs with missing values (e.g. probes not passing quality control) are imputed. Epigenetic ages are then estimated from these partially imputed clock CpGs and used for downstream analyses, e.g. association tests with mortality, age-related illnesses, or other health outcomes. To evaluate how K-NN and PCA imputation affect regression analyses with epigenetic age derived from DNAm with imputed values, we performed Monte Carlo simulations using the GSE264438 chromosome 1 data (581 samples × 24 335 CpGs). First, we simulated an epigenetic age score x once as a linear combination of 500 randomly selected CpGs. Then, for 1000 simulations, we (i) simulated a health outcome y from the linear model y=0.1x+ϵ where ϵ is a vector of independent and identically distributed Gaussian noise scaled to achieve a signal-to-noise ratio of 0.1; (ii) masked the selected CpGs at rates of 20%–70% under missing completely at random (MCAR), missing at random (MAR), and missing not at random (MNAR); and (iii) re-imputed the masked values to obtain the imputed age score $x_{\mathrm{imp}}$ (Rubin 1976). We assessed how well the regression of y on $x_{\mathrm{imp}}$ recovered the true *β*-coefficient by comparing the recovered estimates’ quantiles against ground truth (see Supplementary Methods, available as supplementary data at *Bioinformatics* online for details).

### 2.3 Sliding-window imputation of DNAm EM-seq data

The sliding-window imputation algorithm in slideimp (the *slide_imp* function) processes a whole-genome DNAm dataset chromosome-wise. For each chromosome, the input is an *N* (samples)×*p*_chr_ (number of CpGs on that chromosome) matrix with columns sorted by genomic position.

First, the ordered CpG sites are partitioned into windows of size *w* (base pairs) with an overlap of size *v* between adjacent windows. Optimal *w* and *v* are selected by MC-CV using a single chromosome (e.g. chromosome 22). Additionally, *slide_imp* supports imputing only windows containing a subset of CpGs (i.e. clock CpGs) or creating windows that flank these CpGs by *w* base pairs. For every window, the corresponding submatrix is then imputed using K-NN or PCA imputation. Finally, CpG sites covered by overlapping windows are averaged (Algorithm 1, available as supplementary data at *Bioinformatics* online).

EM-seq DNAm data were processed using the methylKit package (Akalin *et al.* 2012). We compared slideimp’s sliding-window K-NN and PCA imputation against a baseline method that matches sequenced CpG sites to the Illumina EPICv2 A2 manifest followed by K-NN imputation using *impute.knn* (default parameters). Imputation accuracy was assessed using MC-CV by re-imputing the masked beta-values from 5 random participants for each of 1000 randomly selected clock CpGs for 30 replicates. Lastly, to assess the impact of sliding-window imputation on epigenetic age estimates, we examined the partial Pearson’s correlation (adjusted for batch) between chronological age and predicted epigenetic ages using a panel of well-established epigenetic clocks (Hannum *et al.* 2013, Horvath 2013, Horvath *et al.* 2018, Levine *et al.* 2018, Higgins-Chen *et al.* 2022).

## 3 Results

### 3.1 slideimp improves DNAm microarray imputation efficiency and accuracy

**Figure 1:**
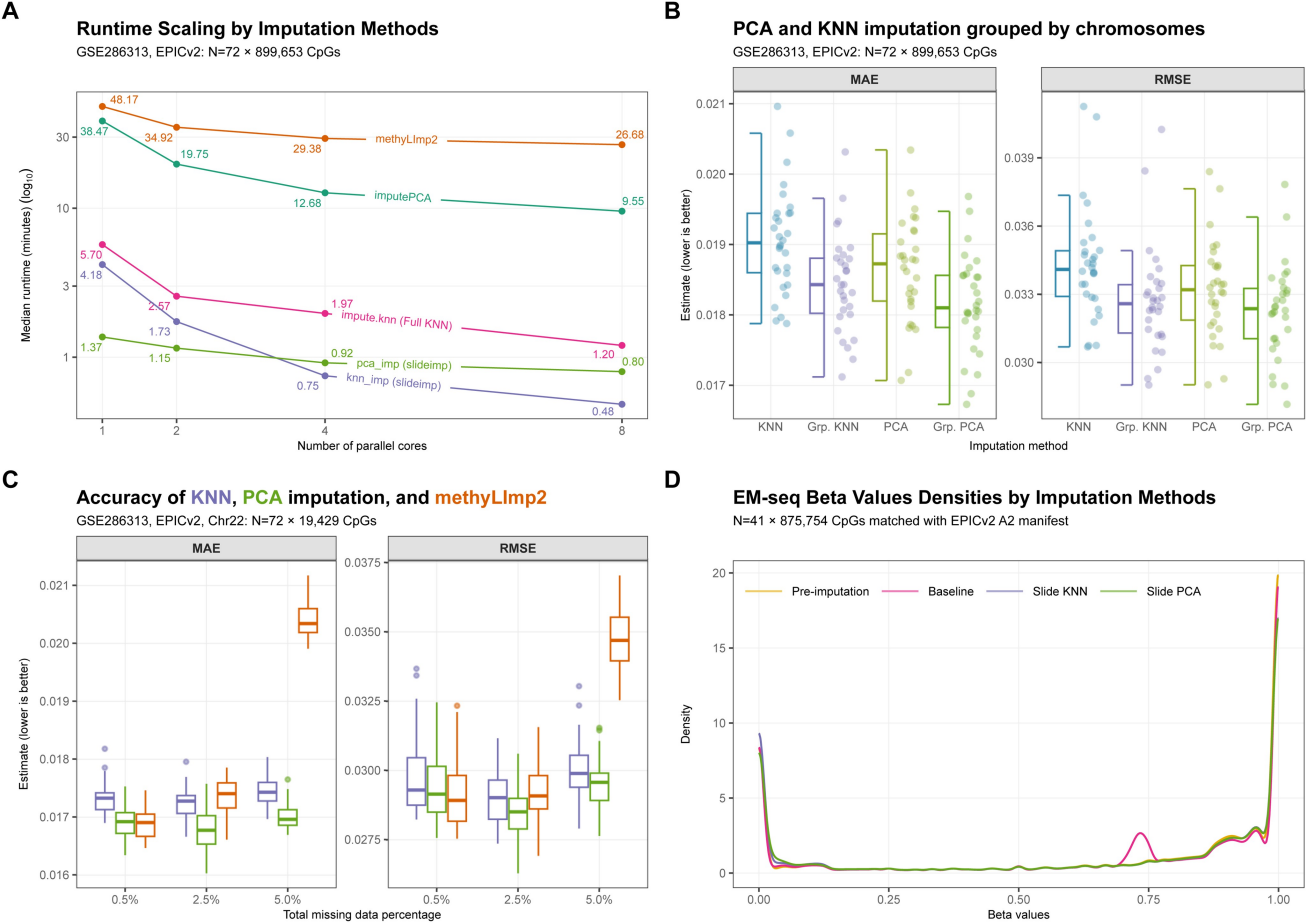
(A) Runtime benchmarks for various imputation methods on the GSE286313 EPICv2 microarray dataset (*N* = 72 × 899 653 CpGs). Points represent the median runtime in minutes across 5 repeats. (B) Imputation accuracy for the full GSE286313 EPICv2 microarray dataset, comparing grouped (by chromosome) versus ungrouped imputation. Grouped imputation demonstrates higher accuracy than ungrouped, with grouped PCA performing best overall. (C) Comparison of imputation accuracy among K-NN, PCA, and *methyLImp2* imputation. At a 0.5% (unmodified) total missingness rate, all methods perform well on these data (GSE286313, EPICv2, *N* = 72, chromosome 22). The accuracy of *methyLImp2* decreases more rapidly as missingness increases. (D) Beta-value densities (*N* = 41 × 875 754 sequenced CpGs matched with the EPICv2 A2 manifest) for the pre-imputed, baseline, and sliding window imputation methods (K-NN and PCA) applied to the EM-seq dataset. Sliding-window imputation produces densities that closely match the pre-imputed data. The baseline method produces a spurious peak at a beta-value of approximately 0.75 due to the default parameters of the *impute.knn* function. PCA: principal component analysis; KNN: K-nearest neighbors; CpG: cytosine–phosphate–guanine; GB: gigabytes; MAE: mean absolute error; RMSE: root mean square error.

Imputing the GSE286313 EPICv2 dataset (*N* = 72 × 899 653 CpGs), *knn_imp* (Algorithm 2, available as supplementary data at *Bioinformatics* online) was ≈12–55× faster than *methyLImp2* and approached 3× faster than *impute.knn* (full K-NN mode) as the number of computing cores increased (Fig. 1A). *pca_imp* (Algorithm 3, available as supplementary data at *Bioinformatics* online) was ≈33–35× faster than *methyLImp2* and ≈12–28× faster than *imputePCA*. This benchmark excluded *impute.knn* with default settings, which performs data pre-partitioning via recursive two-mean clustering with a default cluster size that is too small for DNAm data (see Fig. S1A and S1B, available as supplementary data at *Bioinformatics* online). *knn_imp* ball tree (not shown) was up to 5× faster than *knn_imp* but also yielded less accurate results (Fig. S1B, available as supplementary data at *Bioinformatics* online). Peak memory was reduced with slideimp by ≈3–6× over *methyLImp2* (Fig. S2, available as supplementary data at *Bioinformatics* online). For machines where OpenMP is unavailable, slideimp supports efficient parallelization over chromosomes using mirai (Gao 2026) and bigmemory (Kane *et al.* 2013).

Next, we examined K-NN and PCA imputation grouped by chromosome for full DNAm microarray datasets. For both imputation methods, across both the EPICv2 (Fig. 1B) and MSA datasets (Fig. S3, available as supplementary data at *Bioinformatics* online), the grouped approach was more accurate and faster than the ungrouped approach. This is likely because the information used to impute a CpG site is primarily concentrated proximally to that CpG on the same chromosome (i.e. *in cis*) (Eckhardt *et al.* 2006, Zhang *et al.* 2015, Plaksienko *et al.* 2024).

We benchmarked slideimp’s K-NN and PCA imputation accuracy against *methyLImp2* under increasing missing data rates (Fig. 1C). *methyLImp2* slightly outperformed *pca_imp* at baseline; however, as the missingness rate increased, *methyLImp2*’s accuracy declined more rapidly. This is because *methyLImp2* requires non-missing CpGs to impute missing patterns in other CpGs. Hence, sliding-window K-NN and PCA imputation were more suitable for our EM-seq data where almost every CpG had some missing values.

Figure S4, available as supplementary data at *Bioinformatics* online, shows how K-NN and PCA imputation can affect recovery of the true *β*-coefficient when a simulated health outcome is regressed on epigenetic age derived from partially imputed data. At *N* = 581, a typical sample size for epidemiological studies involving DNAm microarrays, slideimp’s K-NN and PCA imputation yielded unbiased *β*-coefficient estimates with good coverage of ground truth up to 50% per-CpG missingness under MCAR and MAR, with PCA imputation performing best across all scenarios.

### 3.2 Sliding-window imputation of EM-seq data facilitates more accurate epigenetic age estimates

The total missingness rate ranged from 9.7% to 18.6% across chromosomes in our EM-seq dataset. Figure 1D shows the expected bimodal distribution of beta-values from EM-seq DNAm data following sliding-window imputation. In contrast, the baseline method produced a pronounced peak near 0.75. This artifact is caused by the default parameters of *impute.knn* (impute version 1.84.0) whereby CpGs with >50% missing values were imputed using the within-individual mean beta-values across CpGs (rather than the CpG-wise means across individuals).

Sliding-window PCA imputation substantially reduced mean cross-validation MAE from 0.202 to 0.071 and RMSE from 0.313 to 0.102 compared to the baseline method (Table S1, available as supplementary data at *Bioinformatics* online). Furthermore, sliding-window imputation also demonstrated higher precision, reducing the SD across repetitions for both MAE from 0.0053 to 0.0009 and RMSE from 0.0063 to 0.0016.

Across a panel of epigenetic clocks for whole blood, sliding-window PCA substantially increased the correlation between chronological age and epigenetic age: Horvath1 (*r* = 0.121 to *r* = 0.358), Horvath2 (*r* = 0.275 to *r* = 0.523), PhenoAge (*r* = 0.049 to *r* = 0.588), and Hannum (*r* = 0.112 to *r* = 0.513). Correlations between chronological age and epigenetic age derived from PCClocks, epigenetic clocks designed to be more robust to the influence of technical factors and measurement error, also showed substantial increases following sliding-window imputation: PCHorvath2 (*r* = 0.498 to *r* = 0.718), PCHannum (*r* = 0.389 to *r* = 0.719), and PCPhenoAge (*r* = 0.542 to *r* = 0.665) (Table S2, available as supplementary data at *Bioinformatics* online).

## 4 Conclusion

slideimp provides an efficient implementation of K-NN and PCA imputation in R for both microarray and whole-genome DNAm data. slideimp’s sliding-window imputation facilitates the calculation of more accurate epigenetic age estimates from whole-genome DNAm data compared to K-NN imputation using default parameters.

With declining per-sample costs for whole-genome DNAm profiling, and the emergence of long-read sequencing approaches, we anticipate an increased demand for computationally efficient and accurate imputation approaches as implemented in slideimp. Finally, while we demonstrate the utility of slideimp for the imputation of DNAm data, slideimp can be applied to increase imputation efficiency for a variety of other high-dimensional datasets.

## Supplementary Material

btag318_Supplementary_Data

## Data Availability

The DNAm microarray datasets (GSE286313 and GSE264438) are available from the NCBI Gene Expression Omnibus. The EM-seq data can be made available to qualified investigators upon request. The slideimp package is available on CRAN.

## References

[btag318-B1] Akalin A , Kormaksson M, Li S et al methylKit: a comprehensive R package for the analysis of genome-wide DNA methylation profiles. Genome Biol 2012;13:R87. 10.1186/gb-2012-13-10-r87 23034086 PMC3491415

[btag318-B2] Aristizabal MJ , Anreiter I, Halldorsdottir T et al Biological embedding of experience: a primer on epigenetics. Proc Natl Acad Sci USA 2020;117:23261–9. 10.1073/pnas.1820838116 31624126 PMC7519272

[btag318-B3] Curtin RR , Edel M, Shrit O et al mlpack 4: a fast, header-only C++ machine learning library. JOSS 2023;8:5026. 10.21105/joss.05026

[btag318-B4] Eckhardt F , Lewin J, Cortese R et al DNA methylation profiling of human chromosomes 6, 20 and 22. Nat Genet 2006;38:1378–85. 10.1038/ng1909 17072317 PMC3082778

[btag318-B5] Eddelbuettel D, Francois R. Rcpp: Seamless R and C++ Integration, Articles. J Stat Softw 2011;40:1–18. 10.18637/jss.v040.i08

[btag318-B6] Eddelbuettel D, Sanderson C. RcppArmadillo: Accelerating R with high-performance C++ linear algebra. Comput Stat Data Anal 2014;71:1054–63. 10.1016/j.csda.2013.02.005

[btag318-B7] Gao C. *Mirai: Minimalist Async Evaluation Framework for R.* 2026. 10.32614/CRAN.package.mirai (2 March 2026, date last accessed).

[btag318-B8] Goldberg DC , Cloud C, Lee SM et al Scalable screening of ternary-code DNA methylation dynamics associated with human traits. Cell Genom 2025;5:100929. 10.1016/j.xgen.2025.100929 40614726 PMC12534708

[btag318-B9] Hannum G , Guinney J, Zhao L et al Genome-wide methylation profiles reveal quantitative views of human aging rates. Mol Cell 2013;49:359–67. 10.1016/j.molcel.2012.10.016 23177740 PMC3780611

[btag318-B10] Higgins-Chen AT , Thrush KL, Wang Y et al A computational solution for bolstering reliability of epigenetic clocks: implications for clinical trials and longitudinal tracking. Nat Aging 2022;2:644–61. 10.1038/s43587-022-00248-2 36277076 PMC9586209

[btag318-B11] Horvath S. DNA methylation age of human tissues and cell types. Genome Biol 2013;14:R115. 10.1186/gb-2013-14-10-r115 24138928 PMC4015143

[btag318-B12] Horvath S , Oshima J, Martin GM et al Epigenetic clock for skin and blood cells applied to hutchinson gilford progeria syndrome and ex vivo studies. Aging (Albany NY) 2018;10:1758–75. 10.18632/aging.101508 30048243 PMC6075434

[btag318-B13] Josse J , Husson F. missMDA: a package for handling missing values in multivariate data analysis, articles. J Stat Soft 2016;70:1–31. 10.18637/jss.v070.i01

[btag318-B14] Kane M , Emerson JW, Weston S. Scalable strategies for computing with massive data, articles. J Stat Soft 2013;55:1–19. 10.18637/jss.v055.i14

[btag318-B15] Levine ME , Lu AT, Quach A et al An epigenetic biomarker of aging for lifespan and healthspan. Aging (Albany NY) 2018;10:573–91. 10.18632/aging.101414 29676998 PMC5940111

[btag318-B16] Lister R , Pelizzola M, Dowen RH et al Human DNA methylomes at base resolution show widespread epigenomic differences. Nature 2009;462:315–22. 10.1038/nature08514 19829295 PMC2857523

[btag318-B17] Omohundro SM. Five Balltree Construction Algorithms, TR-89-102. Berkeley, California, United States: International Computer Science Institute, 1989.

[btag318-B18] Plaksienko A , Di Lena P, Nardini C et al methyLImp2: faster missing value estimation for DNA methylation data. Bioinformatics 2024;40:btae001. 10.1093/bioinformatics/btae001 38213002 PMC10826905

[btag318-B19] Rubin DB. Inference and missing data. Biometrika 1976;63:581–92. 10.2307/2335739

[btag318-B20] Taudt A , Roquis D, Vidalis A et al METHimpute: imputation-guided construction of complete methylomes from WGBS data. BMC Genomics 2018;19:444. 10.1186/s12864-018-4641-x 29879918 PMC5992726

[btag318-B21] Troyanskaya O , Cantor M, Sherlock G et al Missing value estimation methods for DNA microarrays. Bioinformatics 2001;17:520–5. 10.1093/bioinformatics/17.6.520 11395428

[btag318-B22] Vaisvila R , Ponnaluri VKC, Sun Z et al Enzymatic methyl sequencing detects DNA methylation at single-base resolution from picograms of DNA. Genome Res 2021;31:1280–9. 10.1101/gr.266551.120 34140313 PMC8256858

[btag318-B23] Zhang W , Spector TD, Deloukas P et al Predicting genome-wide DNA methylation using methylation marks, genomic position, and DNA regulatory elements. Genome Biol 2015;16:14. 10.1186/s13059-015-0581-9 25616342 PMC4389802

[btag318-B24] Zhuang BC , Jude MS, Konwar C et al Accounting for differences between Infinium MethylationEPIC v2 and v1 in DNA methylation–based tools. Life Sci Alliance 2025;8:e202403155. 10.26508/lsa.202403155 40628445 PMC12238762

[btag318-B25] Zou LS , Erdos MR, Taylor DL et al BoostMe accurately predicts DNA methylation values in whole-genome bisulfite sequencing of multiple human tissues. BMC Genomics 2018;19:390. 10.1186/s12864-018-4766-y 29792182 PMC5966887

