# Supplementary material for: slideimp: efficient imputation of DNA methylation data
Source: Bioinformatics. 2026 May 21;42(6):btag318. doi: 10.1093/bioinformatics/btag318 (PMC13257852; doi:10.1093/bioinformatics/btag318)
Supplement: btag318_Supplementary_Data [file btag318_supplementary_data.zip › pham et al. 2026 supplement.pdf]

Monte Carlo Simulation of K-NN and PCA Imputation Performance in Epigenetic Clock Regression Under MCAR, MAR, and MNAR Missingness

We investigated how K-NN or PCA imputation affects regression analyses in which chronological age (or similar outcomes) is regressed on epigenetic age estimates derived from the imputed beta matrix measured on the Illumina MSA microarray. We used the GSE264438 MSA microarray chromosome 1 data (N = 581 × 24,335 CpGs).

First, we used sample importance resampling to select 500 random CpGs whose interquartile range distribution matched that of CpGs used by Horvath's DNAm age calculator, yielding a 581×500 matrix **M** (Horvath 2013). We then drew 500 random Gaussian weights **w** and computed true epigenetic clock values as **x**=**Mw**. Then, for each iteration of 1,000 simulations, we generated the outcome **y**=0.1***x**+ε, where ε is random Gaussian noise scaled to achieve a signal-to-noise ratio of 0.1. Next, we introduced missing values into **M** at different rates using `missMethods` (M. S. Santos *et al.* 2019). Under MCAR, values were removed uniformly at random. Under MAR, each CpG j in **M** was paired with a moderately correlated CpG j' ($0.1<$Pearson's $r<0.4$) drawn from the remaining CpGs not in **M**. The missingness in CpG j was made conditional on the observed values of CpG j′ by partitioning the observations at the median of CpG j′, such that the odds of a value being missing in CpG j were twice as high for observations in the group above the median compared to the group below the median (1:2 odds). Under MNAR, the procedure was the same as MAR's, but with the missingness probability vector for each CpG j based on j itself (i.e., $j'=j$). We then imputed **M**, resulting in $\mathbf{M_{imp}}$, estimated the imputed clocks $\mathbf{x_{imp}}=\mathbf{M_{imp}}\mathbf{w}$ and regressed $\mathbf{y}=\beta_{imp}\mathbf{x_{imp}}+\varepsilon$. We compared mean imputation, K-NN imputation, PCA imputation, and ball tree K-NN imputation by comparing the median and 95% quantile interval of the estimated imputed β-coefficient (β_{imp}) and standard error of β_{imp} to the known true quantiles.

Figure S4 shows the coverages of β_{imp} estimates across 1,000 simulations. Estimates using PCA imputation are significantly less biased than those of K-NN imputation under all missing data generating mechanisms, though both methods work well even up to 50% per-CpG missing rate at this dimensionality. The coverage of the respective standard error estimates shows PCA performs better than K-NN even at 20% per-CpG missing rate. Estimates from the K-NN ball tree methods are significantly worse performing than using brute-force K-NN due to the required mean imputation prior to nearest neighbor identification. But at a much lower missing data rate and much higher dimensionality, the trade-off in runtime using K-NN ball tree might be favorable. Overall, PCA imputation performs significantly better in all scenarios in this dataset, but for lower per-CpG missing rates, K-NN provides satisfactory estimates. For both methods, the bias and variance of the estimators increase most under MNAR, followed by MAR, then MCAR, but are reliable at lower per-CpG missing rates.

Reference

Horvath S. DNA methylation age of human tissues and cell types. *Genome Biol* (England) 2013;**14**(10):R115. https://doi.org/10.1186/gb-2013-14-10-r115.

M. S. Santos, R. C. Pereira, A. F. Costa *et al.* Generating Synthetic Missing Data: A Review by Missing Mechanism. *IEEE Access* 2019;**7**:11651–67. https://doi.org/10.1109/ACCESS.2019.2891360.

Algorithm 1 Sliding Window Imputation Implementation

Input: $\mathbf{X} \in \mathbb{R}^{n \times p}$ ▷ n samples × p CpGs, columns sorted by genomic position
Input: $\mathbf{x} \in \mathbb{R}^{p}$ ▷ Sorted genomic positions of the p CpGs
Input: $w > 0$ ▷ Window size (same unit as $\mathbf{x}$)
Input: $0 \le v < w$ ▷ Overlap between consecutive windows (ignored when *flank* = True)
Input: *flank* $\in \{\text{True}, \text{False}\}$ ▷ Whether to use flank mode (Defaults to False)
Input: $\mathcal{S} \subseteq \{1, \ldots, p\}$ ▷ Target column indices (required when *flank* = True)
Input: Imputation method $\mathcal{M} \in$ {K-NN, PCA} with parameter vector $\boldsymbol{\theta}_{\mathcal{M}}$
Output: $\mathbf{X}$ with missing values imputed

```
Step 1: Construct windows
if flank = False then
    Greedily partition columns into windows starting from i = 1:
        Window t spans [x_i, x_i + w), collecting all CpGs j with x_j in that range.
        Record start/end column indices a_t, b_t.
        Advance i to the first CpG with position ≥ x_i + w − v.
        Stop once the window covers column p. Let T be the last window index.
else
    For each target s ∈ S, build one window t collecting all CpGs j with |x_j − x_s| < w.
        Record start/end column indices a_t, b_t, and local index ℓ_t = s − a_t + 1.
        Let T = |S|.
end if
Step 2: Compute overlap counts
if flank = False then
    c ∈ Z^p,  c_j ← Σ_t 1[a_t ≤ j ≤ b_t]   for each j = 1, ..., p
end if
Step 3: Window-wise imputation
R ← 0_{n×p}                                      ▷ Accumulator matrix
for t = 1, ..., T do
    W_t ← X[ , a_t : b_t]                         ▷ Extract window submatrix
    if M is K-NN then
        W̃_t ← K-NN-Impute(W_t, θ_K-NN)
    else if M is PCA then
        W̃_t ← PCA-Impute(W_t, θ_PCA)
    end if
    if flank = False then
        R[ , a_t : b_t] ← R[ , a_t : b_t] + W̃_t   ▷ Accumulate all imputed columns
    else
        R[ , a_t + ℓ_t − 1] ← W̃_t[ , ℓ_t]         ▷ Store only the target column
    end if
end for
Step 4: Finalize
if flank = False then
    for j = 1 to p do
        X[ , j] ← R[ , j]/c_j
    end for
else
    for j ∈ S do
        X[ , j] ← R[ , j]
    end for
end if
return X
```

Algorithm 2 Brute-force K-NN Imputation Implementation (modified from Troyanskaya et al., 2001)

Input: $\mathbf{X} \in \mathbb{R}^{n \times p}$ ▷ n samples × p CpG sites
Input: K ▷ Number of nearest neighbors
Input: α ▷ Distance power for weighting
Input: *cores* ▷ Number of parallel cores
Output: $\mathbf{X}$ with missing values imputed

```
X_M ← CpGs of X with any missing values
for each CpG site j in X_M in parallel across cores do
    T_j ← ∅                                         ▷ Running top-K neighbors, sorted by d_{j,l}
    bound ← ∞                                       ▷ Worst d_{j,l} among current top-K
    for each CpG site l in X, l ≠ j do
        O_{j,l} ← {i : X_ij and X_il are both observed}
        Accumulate d_{j,l} ← Σ_{i∈O_{j,l}} (X_ij − X_il)^2, with early exit if d_{j,l} exceeds bound
        if |T_j| < K or d_{j,l} < bound then
            Insert (l, d_{j,l}) into T_j, keeping only the K smallest
            bound ← largest d_{j,l} in T_j           ▷ Tightens as better neighbors are found
        end if
    end for
    N_j ← indices in T_j
    ε ← 10^{-10}
    w_l ← 1 / (d_{j,l} + ε)^α   ∀ l ∈ N_j           ▷ Inverse-distance weights
    for each sample i = 1 to n do
        if X_ij is missing then
            X_ij ← (Σ_{l∈N_j} w_l X_il) / (Σ_{l∈N_j} w_l)
        end if
    end for
end for
Apply column-mean imputation for any remaining missing values   ▷ Fallback
return X
```

Where: $\mathcal{O}_{j,l} \leftarrow \{i : X_{ij} \text{ and } X_{il} \text{ are both observed}\}$; $d_{j,l} \leftarrow \sum_{i \in \mathcal{O}_{j,l}} (X_{ij} - X_{il})^2$; $w_l \leftarrow \frac{1}{(d_{j,l} + \epsilon)^\alpha} \quad \forall\, l \in \mathcal{N}_j$; $X_{ij} \leftarrow \frac{\sum_{l \in \mathcal{N}_j} w_l X_{il}}{\sum_{l \in \mathcal{N}_j} w_l}$

Algorithm 3 Regularized Iterative PCA Imputation (modified from Josse and Husson, 2016)

Input: $\mathbf{X}, \mathbf{w}$ ▷ Input matrix (n samples × p CpG sites) and optional row weights ($\sum_i w_i = 1$)
Input: S, γ, *init*, ε ▷ Components to retain, ridge coefficient, initialization mode, convergence threshold
Output: $\mathbf{X}$ with missing values imputed

$K \leftarrow S+1$ ▷ One extra eigenpair retained to cap $\hat{\sigma}^2$ at λ_{S+1}
$\mathbf{W} \leftarrow$ indicator matrix where $W_{ij} = 1$ if X_{ij} is observed, 0 otherwise
▷ Initial weighted column means and standard deviations from observed entries only

$$\mu_j^{(0)} \leftarrow \frac{\sum_i w_i W_{ij} X_{ij}}{\sum_i w_i W_{ij}}, \quad \eta_j^{(0)} \leftarrow \sqrt{\frac{\sum_i w_i W_{ij} X_{ij}^2}{\sum_i w_i W_{ij}} - (\mu_j^{(0)})^2}$$

$\widetilde{X}_{ij}^{(0)} \leftarrow (X_{ij} - \mu_j^{(0)})/\eta_j^{(0)}$ for observed (i,j)
if *init* $= 0$ **then**
  $\widetilde{X}_{ij}^{(0)} \leftarrow 0$ for missing (i,j) ▷ Column-mean initialization (standardized mean is 0)
else
  $\widetilde{X}_{ij}^{(0)} \sim \mathcal{N}(0,1)$ for missing (i,j) ▷ Random initialization in standardized space
end if
$\ell \leftarrow 0, \quad \hat{\mathbf{X}}^{(0)} \leftarrow \widetilde{\mathbf{X}}^{(0)}, \quad \mathcal{L}^{(0)} \leftarrow \infty$ ▷ Seed for first re-imputation
▷ $\widetilde{\mathbf{X}}^{(\ell)}$: completed centered/scaled matrix. $\hat{\mathbf{X}}^{(\ell)}$: its rank-$S$ PCA reconstruction.
repeat
  $\ell \leftarrow \ell + 1$

  ▷ Step (2.a): Re-impute missing and restandardize affected columns
  $\widetilde{X}_{ij}^{(\ell)} \leftarrow \hat{X}_{ij}^{(\ell-1)}$ for all missing (i,j)
  Recompute $\mu_j^{(\ell)}$, $\eta_j^{(\ell)}$ using the updated column and rescale:
   $\widetilde{X}_{ij}^{(\ell)} \leftarrow \big(\widetilde{X}_{ij}^{(\ell)}\, \eta_j^{(\ell-1)} + \mu_j^{(\ell-1)} - \mu_j^{(\ell)}\big)/\eta_j^{(\ell)}$

  ▷ Step (2.b): Truncated SVD via the smaller Gram matrix
  $\mathbf{Y} \leftarrow \operatorname{diag}(\sqrt{\mathbf{w}})\, \widetilde{\mathbf{X}}^{(\ell)}$
  if $n \geq p$ **then** ▷ Tall: work with the $p \times p$ Gram
    $\mathbf{G} \leftarrow \mathbf{Y}^\top \mathbf{Y} \in \mathbb{R}^{p \times p}$
    Eigendecomposition of $\mathbf{G}$ yielding its top K eigenpairs $(\lambda_s^{(\ell)}, \mathbf{v}_s^{(\ell)})_{s=1}^K$
    $\mathbf{U}^{(\ell)} \leftarrow \operatorname{diag}(1/\sqrt{\mathbf{w}})\, \mathbf{Y}\, \mathbf{V}^{(\ell)} \operatorname{diag}\big(1/\sqrt{\lambda_s^{(\ell)}}\big)$
  else ▷ Wide: work with the $n \times n$ Gram
    $\mathbf{G} \leftarrow \mathbf{Y}\, \mathbf{Y}^\top \in \mathbb{R}^{n \times n}$
    Eigendecomposition of $\mathbf{G}$ yielding its top K eigenpairs $(\lambda_s^{(\ell)}, \tilde{\mathbf{u}}_s^{(\ell)})_{s=1}^K$
    $\mathbf{V}^{(\ell)} \leftarrow \mathbf{Y}^\top\, \widetilde{\mathbf{U}}^{(\ell)} \operatorname{diag}\big(1/\sqrt{\lambda_s^{(\ell)}}\big)$
    $\mathbf{U}^{(\ell)} \leftarrow \operatorname{diag}(1/\sqrt{\mathbf{w}})\, \widetilde{\mathbf{U}}^{(\ell)}$
  end if

  ▷ Step (2.c): Regularized rank-S reconstruction
  ▷ Tail eigenvalue sum via trace identity: $\sum_{s=S+1}^{\min(n,p)} \lambda_s^{(\ell)} = \operatorname{tr}(\mathbf{G}) - \sum_{s=1}^{S} \lambda_s^{(\ell)}$

$$(\hat{\sigma}^2)^{(\ell)} \leftarrow \min\left(\gamma \cdot \frac{\operatorname{tr}(\mathbf{G}) - \sum_{s=1}^{S} \lambda_s^{(\ell)}}{(n-S)(p-S)}, \lambda_{S+1}^{(\ell)}\right)$$

$$\hat{X}_{ij}^{(\ell)} \leftarrow \sum_{s=1}^{S} \left(\sqrt{\lambda_s^{(\ell)}} - \frac{(\hat{\sigma}^2)^{(\ell)}}{\sqrt{\lambda_s^{(\ell)}}}\right) u_{is}^{(\ell)}\, v_{js}^{(\ell)}$$ ▷ Rank-S low-rank approximation of $\widetilde{\mathbf{X}}^{(\ell)}$

  $\mathcal{L}^{(\ell)} \leftarrow \sum_{i,j} w_i W_{ij} \big(\widetilde{X}_{ij}^{(\ell)} - \hat{X}_{ij}^{(\ell)}\big)^2$ ▷ Weighted objective
until $\big|1 - \mathcal{L}^{(\ell)}/\mathcal{L}^{(\ell-1)}\big| \leq \varepsilon$
return $X_{ij} \leftarrow \widetilde{X}_{ij}^{(\ell)} \eta_j^{(\ell)} + \mu_j^{(\ell)}$ for missing (i,j) ▷ Final unstandardization

Table S1

Method	Optimal Parameters	MAE		RMSE	
		Mean	SD	Mean	SD
Baseline	—	0.2019	0.0053	0.3133	0.0063
SW KNN	Window Size: 10,000 bp; Overlap Size: 1,000 bp; N Neighbors: 20; Distance Power: 2.25;	0.0729	0.0012	0.1102	0.0019
SW PCA	Window Size: 15,000 bp; Overlap Size: 1,000 bp; N Components: 7; Ridge Coefficient: 1.5;	0.0711	0.0009	0.1017	0.0016

Table S1. Cross-validation errors of baseline vs sliding window imputation methods. Imputation accuracy was assessed via MC-CV by randomly masking beta-values from 5 participants across 1,000 clock CpGs over 30 independent replicates. Optimal parameters were chosen based on combinations with the lowest mean RMSE. For both MAE and RMSE, the sliding window methods significantly reduced cross-validation errors compared to baseline. MAE: Mean Absolute Error; RMSE: Root Mean Square Error; SW: sliding window; KNN: k-nearest neighbors; PCA: principal component analysis; MC-CV: Monte Carlo cross-validation; bp: base pairs.

Table S2

Method	CpG-based Clocks				PC-based Clocks			
	Horvath1	Horvath2	PhenoAge	Hannum	PCHorvath1	PCHorvath2	PCHannum	PCPhenoAge
Baseline	0.121	0.275	0.049	0.112	0.758	0.498	0.389	0.542
SW KNN	0.424	0.626	0.610	0.522	0.797	0.732	0.749	0.679
SW PCA	0.358	0.523	0.588	0.513	0.795	0.718	0.719	0.665

Table S2. Batch-adjusted partial Pearson's correlation between chronological ages and a panel of epigenetic clocks estimated from beta values imputed by baseline vs. sliding window methods. The CpG-based clocks showed the greatest increases in partial correlation values. The PC Clocks are more robust to technical and measurement errors and showed smaller increases, but still showed substantial increases. CpG: cytosine–phosphate–guanine; PC: Principal component; SW: sliding window; KNN: k-nearest neighbors; PCA: principal component analysis.

Figure S1. Imputation accuracy comparison across KNN−based methods

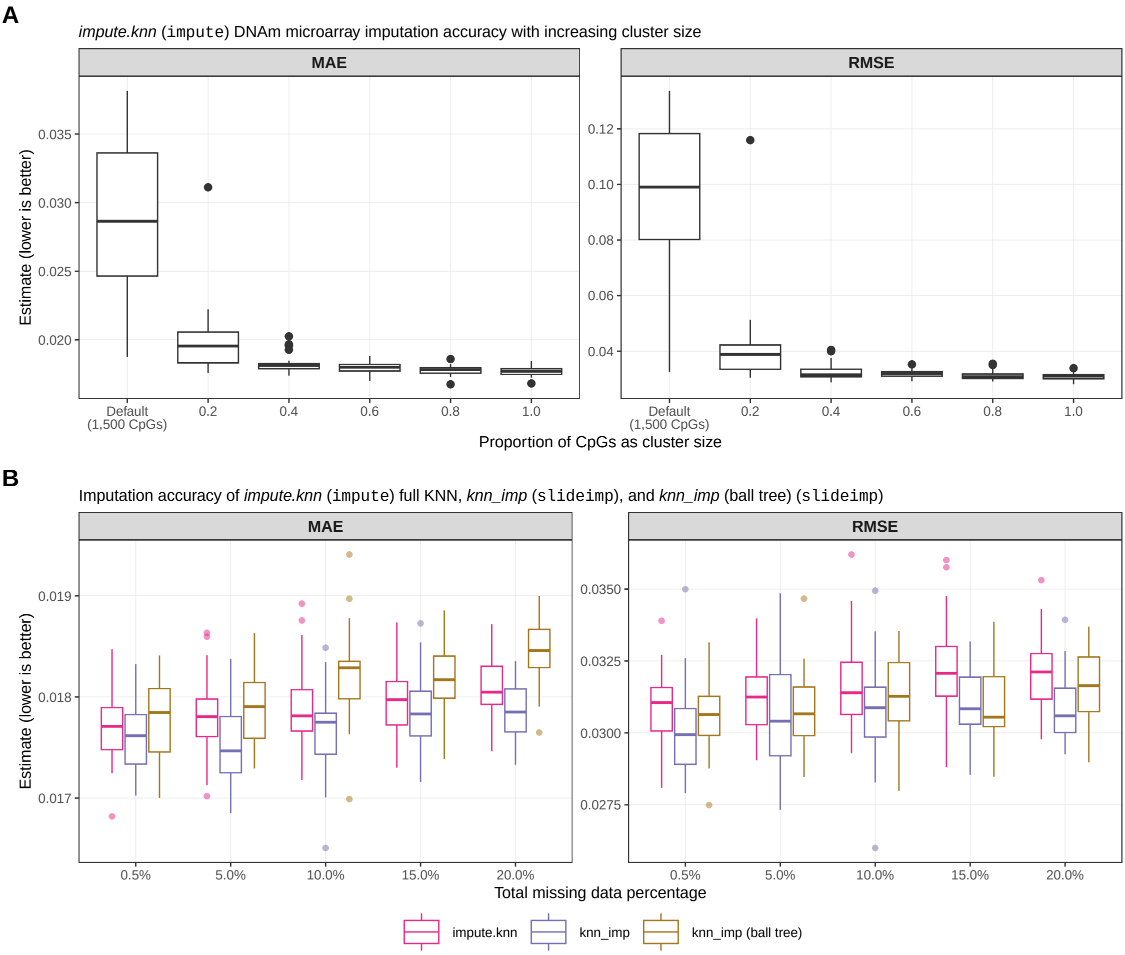

Figure S1. Imputation accuracy of KNN−based methods evaluated using GSE286313 EPICv2 chromosome 22 data (N=72; 19,429 CpGs) via Monte−Carlo cross−validation by imputing 5,000 randomly masked values for 30 replicates **(A)** Impact of cluster size on *impute.knn* (`impute`) accuracy. *impute.knn* utilizes recursive two−mean clustering to reduce computational load; however, the default cluster size (1,500 CpGs) is too small for typical DNAm microarrays, leading to very poor imputation accuracy and precision. Peak performance is only achieved using 'full KNN' (proportion = 1.0, i.e., 19,429 CpGs). **(B)** Comparative accuracy across increasing total data missingness rates. By using weighted distance averaging, the *knn_imp* function (`slideimp`) consistently outperforms the *impute.knn* across all tested missingness levels. The *knn_imp* ball tree becomes increasingly less accurate with increasing missing rate as well as performing worst at all missingness levels due to the required mean imputation prior to neighbor search. N: Sample size; KNN: k−nearest neighbors; DNAm: DNA methylation; CpG: cytosine–phosphate–guanine; MAE: Mean Absolute Error; RMSE: Root Mean Square Error.

Figure S2. Peak Memory Usage Scaling by Imputation Methods

GSE286313, EPICv2: N=72 × 899,653 CpGs

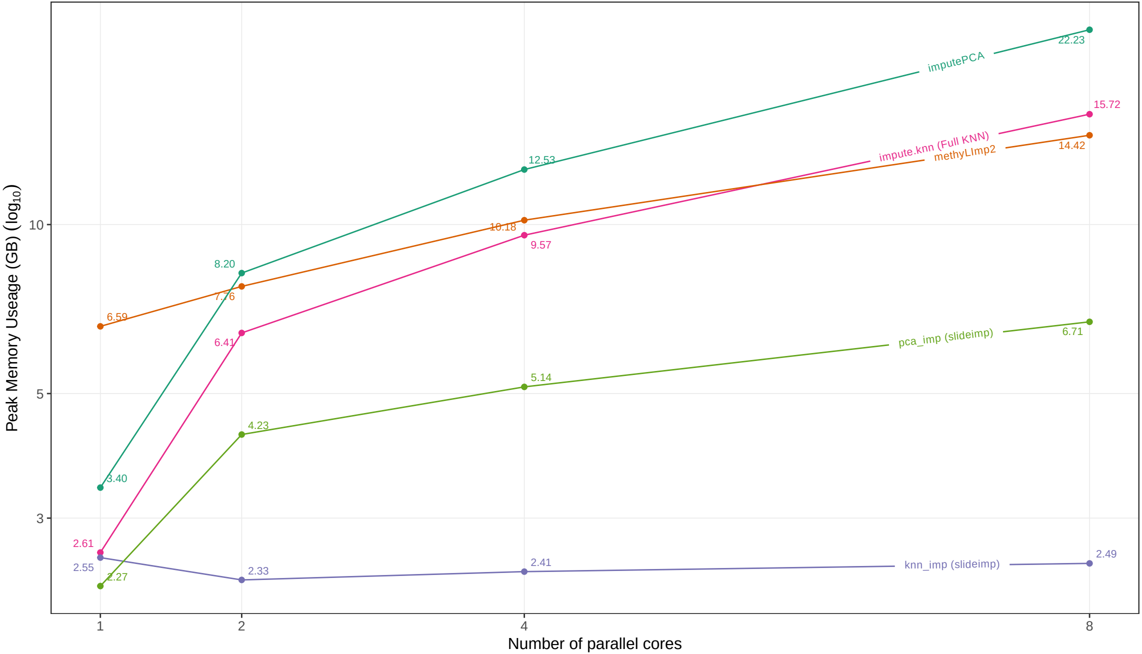

Figure S2. Peak memory usage benchmarks for various imputation methods on the GSE286313 EPICv2 microarray dataset (N = 72; 899,653 CpGs). Points represent the median peak memory usage in gigabytes across 5 repeats. N: Sample size; PCA: principal component analysis; KNN: k−nearest neighbors; GB: gigabytes; CpG: cytosine–phosphate–guanine.

Figure S3. PCA and KNN imputation grouped by chromosomes

GSE264438, MSA, multi−tissue samples: N=581 × 271,613 CpGs

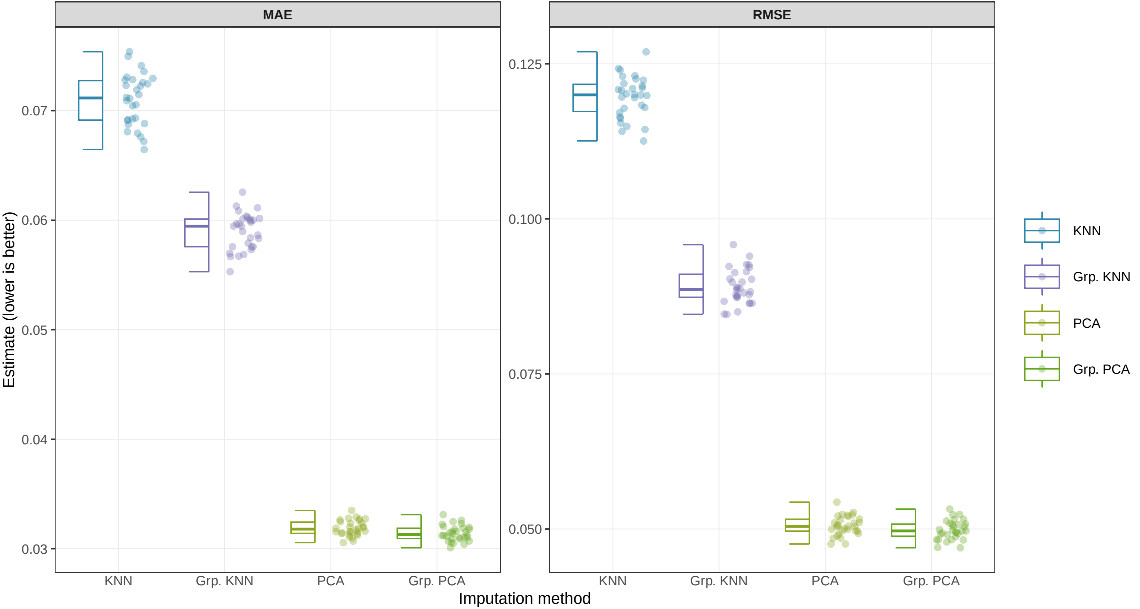

Figure S3. Imputation accuracy for the full GSE264438 MSA microarray dataset (N = 581; 271,613 CpGs) comparing grouped (by chromosome) versus ungrouped imputation. Grouped imputation shows higher accuracy, with grouped PCA performing best overall. PCA outperformed KNN for this dataset because extreme tissue heterogeneity dominates the variance in these data; this variance is more effectively captured by the low−rank approximation of PCA imputation regardless of grouping leading to much higher accuracy for the PCA imputation method. N: Sample size; PCA: principal component analysis; KNN: k−nearest neighbors; CpG: cytosine–phosphate–guanine; MAE: Mean Absolute Error; RMSE: Root Mean Square Error.

Figure S4. Monte Carlo Simulation of KNN and PCA Imputation under MCAR, MAR, MNAR

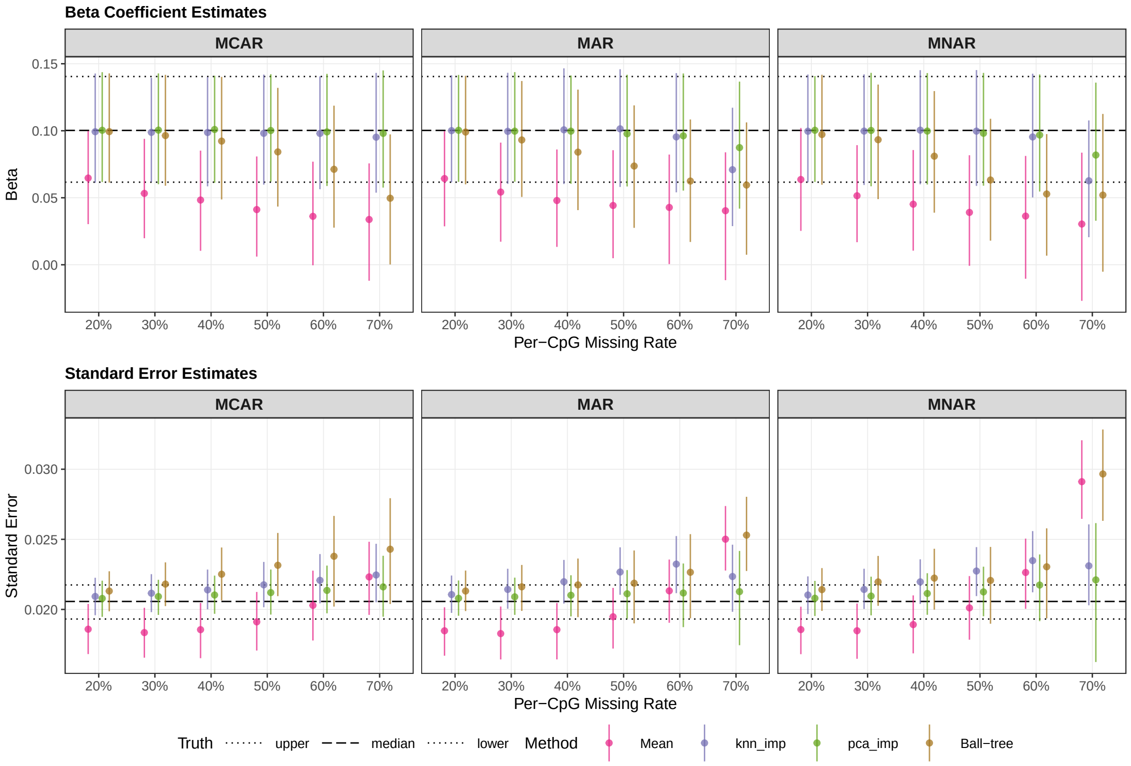

Figure S4. Results from 1000 Monte Carlo simulations of regression estimates under MCAR, MAR, and MNAR mechanisms across various per−CpG missing rates. For 1000 simulations, a simulated health outcome **y** was regressed on the imputation of simulated clock **x** generated from 500 randomly sampled CpGs using the GSE264438 data (N=581; 24,335 CpGs) at a known beta coefficient value of 0.1 and signal−to−noise ratio of 0.1. Beta coefficient estimates are shown in the upper panels, and standard error estimates of the beta coefficient in the lower panels. Points represent the medians and lines represent the 95% quantile intervals of the distributions. Dotted and dashed lines represent the ground truth quantiles. Overall, PCA imputation outperforms KNN due to higher prediction accuracy (Figure S3). Mean and KNN ball tree results are biased. The quantile distributions of both methods sufficiently cover the true scenarios at lower missing rates, but PCA performs especially well at higher missing rates under both MCAR and MAR. N: Sample size; PCA: principal component analysis; KNN: k−nearest neighbors; CpG: cytosine–phosphate–guanine; MCAR: Missing Completely at Random; MAR: Missing at Random; MNAR: Missing Not at Random.
